# Cancer risk associated with DPP4 inhibitors in type 2 diabetes: A pharmacovigilance analysis of the FDA Adverse Event Reporting System (FAERS)

**DOI:** 10.1371/journal.pone.0345281

**Published:** 2026-03-20

**Authors:** Wan Xiong, Yilin Li, Juanjuan Huang, Gefei He, Ji Sun

**Affiliations:** 1 Department of Pharmacy, The First Hospital of Changsha, Changsha, China; 2 Department of Pharmacy, The Affiliated Changsha Hospital of Xiangya School of Medicine, Central South University, Changsha, China; 3 Department of Information and Digital Technology, PowerChina Zhongnan Engineering Corporation Limited, Changsha, China; Pelita Harapan University Faculty of Medicine: Universitas Pelita Harapan Fakultas Kedokteran, INDONESIA

## Abstract

**Background:**

The use of dipeptidyl peptidase 4 (DPP4) inhibitors in treating type 2 diabetes mellitus (T2DM) is increasingly widespread. However, their association with malignancy risk has not been comprehensively evaluated in real-world clinical settings. This study, utilizing the Food and Drug Adverse Event Reporting System (FAERS) database, investigated the potential link between DPP4 inhibitors and malignancies.

**Methods:**

Data from the FAERS database (January 2019 to December 2024) were analyzed. Descriptive statistics assessed patient demographics for each drug-event combination, while disproportionality analysis based on Reporting Odds Ratio (ROR) and Information Component (IC) metrics valuated cancer-related adverse event (AE) risks. Binary logistic regression was used to minimize potential bias.

**Results:**

A comprehensive analysis identified 3,764 AE reports linked to malignancies in T2DM individuals treated with DPP4 inhibitors. Signal detection analysis revealed that sitagliptin exhibited the strongest and most extensive positive signals across both the Preferred Terms and Standardized Medical Dictionary for Regulatory Activities Queries. Specifically, significant signals were observed for malignancies (ROR_025_: 13.80, IC_025_: 3.48), tumor lysis syndrome (ROR_025_: 5.79, IC_025_: 2.32), and site-specific neoplasms (e.g., uterine, fallopian tube, and prostate). Age distribution analysis indicated that the median age of individuals reporting malignancy-related AEs exceeded 70 years in most drug groups. Furthermore, in studies with larger sample sizes, the median time to AE onset for for DPP4 inhibitors ranged from 13 to 15 months.

**Conclusions:**

This study demonstrates significant associations between all five DPP4 inhibitors and malignancy-related AEs, with sitagliptin showing the highest risk profile. These findings highlight the need for further validation through large-scale prospective studies to verify the observed pharmacovigilance signals and to elucidate their potential clinical significance and underlying biological mechanisms.

## Introduction

Type 2 diabetes mellitus (T2DM) is a complex chronic disorder caused by multiple factors. Its global prevalence continues to rise, currently affecting over 600 million people and significantly burdening healthcare systems [1]. Among available treatments, dipeptidyl peptidase 4 (DPP4) inhibitors, a class of oral hypoglycemic agents that enhance incretin activity, have been increasingly used in clinical practice over the past decade. Currently, the Food and Drug Administration (FDA) approved DPP4 inhibitors, including sitagliptin, saxagliptin, linagliptin, alogliptin, and vildagliptin, are favored for their low risk of severe hypoglycemia and neutral effect on body weight [2]. These benefits stem from DPP4’s central role in glucose metabolism, in which it degrades incretins such as glucagon-like peptide-1 (GLP-1). GLP-1 receptors are not limited to glucose-regulating tissues but are also found in the thyroid, pancreas, brain, kidneys, and bones, suggesting potential effects beyond glycemic control [3]. This broader physiological impact has attracted considerable research attention, particularly in exploring the link between DPP4 inhibition and conditions like pancreatitis and pancreatic cancer in individuals with T2DM, making it a compelling area of study [4].

However, despite extensive investigations, the relationship between DPP4 inhibitors and cancer risk remains inconclusive, with ongoing debates about their safety profile. Early studies analyzing FDA reports identified an adverse event report signal (signal defined as the lower limit of the 95% confidence interval of the ROR being greater than 1), which indicated a potential association between DPP4 inhibitor exposure and pancreatic cancer development [5]. Additionally, it has been proposed that DPP4 inhibitors might induce α-cell hyperplasia, increasing cellular proliferation and dysplasia, potentially progressing to neuroendocrine tumors [6]. A population-based cohort study utilizing the Clinical Practice Research Datalink found that the use of DPP4 inhibitors may be associated with an increased risk of cholangiocarcinoma in adults with T2DM, compared to the use of other second- or third-line antidiabetic medications [7]. A systematic review further elucidated the dual role of DPP4 in female cancers, indicating that it may influence cancer progression through modulation of immune responses and cell migration, while its inhibitors (e.g., sitagliptin) may exert opposing effects in different cancer types [8]. Additionally, several of these studies indicated that while DPP4 inhibitors significantly increased the risk of pancreatitis, they did not elevate pancreatic cancer risk [9,10].

While standard clinical trials are often underpowered to detect rare, delayed-onset AEs like malignancy due to limited sample sizes and follow-up durations, spontaneous reporting systems such as the FAERS containing over a million post-marketing reports provide a critical complementary approach. The demonstrated reliability of FAERS in identifying novel and rare AEs, particularly through its comprehensive capture of real-world medication use across diverse populations, makes it uniquely valuable for pharmacovigilance research [11]. Based on these considerations, our study systematically evaluated FAERS data to investigate potential associations between DPP4 inhibitors and malignancy risk, thereby contributing to a more comprehensive understanding of their risk-benefit profile.

## Materials and methods

### Data sources

A pharmacovigilance analysis was conducted using DPP4 inhibitor-associated AE reports from the FAERS database between the first quarter of 2019 (2019Q1) and the fourth quarter of 2024 (2024Q4). The FAERS database is a publicly accessible global pharmacovigilance database containing fully anonymized data without personal identifiers. It comprises seven core data files: DEMO (demographic and administrative information), INDI (indications for use/diagnosis), DRUG (drug details), REAC (coded adverse events), OUTC (patient outcomes), THER (therapy start and end dates), and RPSR (report sources). AEs are coded using Preferred Terms (PTs) from the Medical Dictionary for Regulatory Activities (MedDRA), with hierarchical classification achieved through High-Level Terms (HLTs), High-Level Group Terms (HLGTs), and System Organ Classes (SOCs). Additionally, Standardized MedDRA Queries (SMQs) can be utilized for clustering analysis of specific medical conditions. While this rich dataset enables large-scale safety analyses, it is important to note that as a spontaneous reporting system, FAERS data are subject to well-documented limitations including underreporting, reporting bias, and variable data completeness [11,12]. These inherent characteristics preclude definitive causal inference but remain valuable for signal detection. In this study, we systematically extracted and analyzed these data to evaluate the potential association between DPP4 inhibitors and malignancy risk.

### Procedures

**Fig 1:**
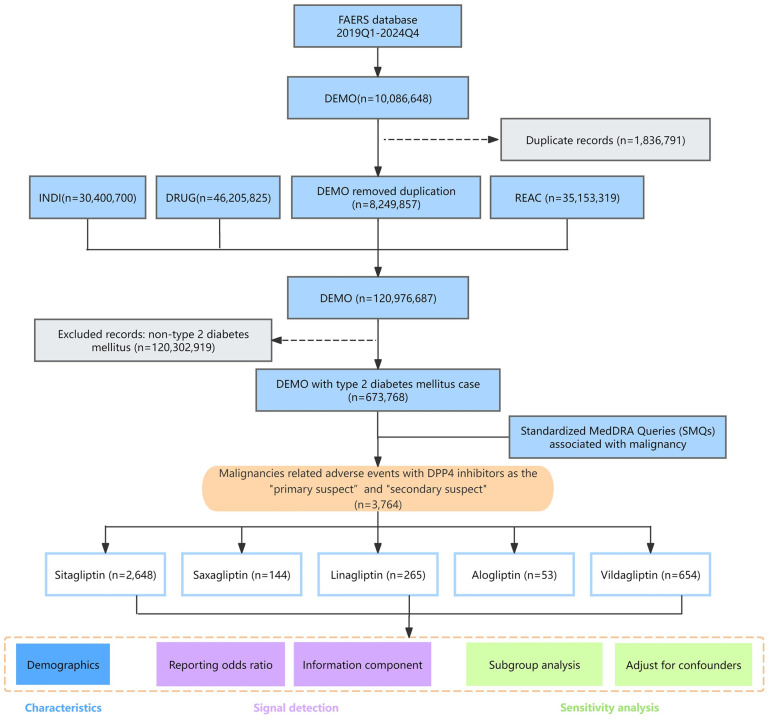
The flow chart of the study. Abbreviations: DEMO, demographic and administrative information; INDI, indications for use/diagnosis; DRUG, drug details; REAC, coded adverse events.

The 10,086,648 raw reports from FAERS, spanning from 2019Q1 to 2024Q4, were imported into a MySQL database, after which data cleaning and integration were performed using Python pandas (version 2.1.0). To ensure data integrity, duplicate reports were eliminated following FDA recommended variable matching methods [13]. Specifically, duplicate cases were removed based on the delete file package, retaining the most recent FDA_DT and higher PRIMARYID when CASEIDs were identical. This process resulted in the exclusion of 1,836,791 duplicate reports (Fig 1).

To control for confounding factors, the analysis was restricted to individuals with T2DM, resulting in the inclusion of 673,768 valid reports. The analysis focused on five DPP4 inhibitors: sitagliptin, saxagliptin, linagliptin, alogliptin, and vildagliptin. The role of AEs was assigned by reporters (e.g., healthcare professionals, patients, or manufacturers) using specific role codes, including primary suspect (PS), secondary suspect (SS), concomitant (C), and interacting (I). To enhance data accuracy and obtain better signal intensity, cases with the report role code of PS and SS were included in the analysis.

AE analysis was conducted using the MedDRA terminology system. The analysis was performed at two levels: PTs related to malignancy were retrieved at the SOC level (code: 10029104). Additionally, relevant PTs were also retrieved based on 9 SMQs, which included malignancies; breast neoplasms, malignant and unspecified; premalignant disorders; skin neoplasms, malignant and unspecified; ovarian neoplasms, malignant and unspecified; malignant lymphomas; prostate neoplasms, malignant and unspecified; uterine and fallopian tube neoplasms, malignant and unspecified and tumour lysis syndrome. Through stratified analysis, the study systematically assessed the cancer risks associated with DPP4 inhibitors.

### Statistical analysis

To evaluate the potential patterns of AEs, we conducted a systematic analysis of patient demographic characteristics and time to onset. In terms of data analysis methods, categorical variables were described using frequencies and percentages, while continuous variables were expressed as medians and interquartile ranges.

For signal detection of DPP4 inhibitor-associated malignancies versus other T2DM treatments, we conducted disproportionality analysis using complementary methods: the ROR for interpretable odds ratios and the IC with Bayesian modeling for robust sparse-data analysis. [14]. A signal was considered confirmed only when both of the following conditions were satisfied: (1) the lower limit of the 95% confidence interval (CI) for ROR (ROR_025_) was greater than 1; and (2) the lower limit of the 95%CI for IC (IC_025_) was greater than 0 (see S1 Table for formulas) [12].

Given that T2DM individuals often receive combination therapies of glucose-lowering agents with different mechanisms of action, and frequently experience polypharmacy due to multiple comorbidities, such combination drug use may influence the risk of malignancy development and progression. To enhance the robustness of the results and control for potential confounding factors, we utilized a binary logistic regression model to adjust for variables such as age, sex, and concomitant medication use. To identify concomitant medications for adjustment, we first selected the top 10 most frequent concomitant drugs based on FAERS frequency, then supplemented this with a review of their FDA labels for documented cancer risks. Only those with identified risks were included as binary covariates. All data analyses were independently performed by two researchers. Data extraction was conducted using MySQL 8.0, data cleaning and integration executed using Python pandas (v2.1.0), and statistical analysis was performed using Excel and SPSS 27.0.

## Results

### Clinical characteristics

Based on analysis of FAERS database reports from 2019Q1 to 2024Q4, we identified 3,764 malignancy-related adverse events associated with DPP4 inhibitors. Statistical analysis revealed sitagliptin was associated with the highest number of spontaneous reports among DPP4 inhibitors, followed by vildagliptin, linagliptin, saxagliptin and alogliptin (Table 1).

**Table 1:** Demographic information on malignancy risks with DPP4 inhibitors.

	Cases, N (%)				
Characteristic	Sitagliptin	Saxagliptin	Linagliptin	Alogliptin	Vildagliptin
**Total cases**	2,648	144	265	53	654
**Gender**					
**Data available**	2,490	127	252	45	596
**Female**	1,188 (47.71%)	59 (46.46%)	130 (51.59%)	17 (37.78%)	282 (47.32%)
**Male**	1,302 (52.29%)	68 (53.54%)	122 (48.41%)	28 (62.22%)	314 (52.68%)
**Age (years)**					
**0-19**	0	0	0	0	0
**20-29**	3 (0.18%)	0	3 (1.35%)	0	0
**30-39**	18 (1.09%)	0	0	0	0
**40-49**	27 (1.63%)	0	13 (5.83%)	4 (8.89%)	8 (1.43%)
**50-59**	195 (11.77%)	33 (28.95%)	27 (12.11%)	7 (15.56%)	38 (6.8%)
**60-69**	488 (29.45%)	51 (44.74%)	37 (16.59%)	6 (13.33%)	160 (28.62%)
**70-79**	574 (34.64%)	22 (19.3%)	65 (29.15%)	18 (40%)	198 (35.42%)
**80-89**	327 (19.73%)	8 (7.02%)	73 (32.74%)	7 (15.56%)	148 (26.48%)
**90-100**	25 (1.51%)	0 (0%)	5 (2.24%)	3 (6.67%)	7 (1.25%)
**Data available**	1,657	114	223	45	559
**Median (IQR)**	70.37 (63-78)	67 (55.64-71)	73 (62-82)	71 (60-78)	71 (64-80)
**Reported countries (Top 3)**					
**1**	FR 981 (37.05%)	US 60 (41.67%)	US 83 (31.32%)	JP 30 (56.60%)	FR 281(42.97%)
**2**	US 954 (36.03%)	FR 49 (34.03%)	JP 32 (12.08%)	KR 12 (22.64%)	JP 136 (20.80%)
**3**	JP 132 (4.98%)	JP 19 (13.19%)	BE 18 (6.79%)	GB 6 (11.32%)	PT 113 (17.28%)
**Outcomes**					
**Data available**	5,242	274	420	100	1,081
**Hospitalized (HO)**	1,697 (32.37%)	67 (24.45%)	142 (33.81%)	23 (23.00%)	415 (38.39%)
**Disabled (DS)**	61 (1.16%)	11 (4.01%)	5 (1.19%)	0	29 (2.68%)
**Life threating (LT)**	663 (12.65%)	43 (15.69%)	32 (7.62%)	10 (10.00%)	267 (24.70%)
**Died (DE)**	1,065 (20.32%）	63 (22.99%)	34 (8.10%)	14 (14.00%)	32 (2.96%)
**Other outcomes (OT）**	1,756 (33.50%)	90 (32.85%)	207 (49.29%)	53 (53.00%)	338 (31.27%)

Abbreviations: FR, France; US, United States; JP, Japan; KR, South Korea; BE, Belgium; GB, United Kingdom; PT, Portugal; IQR, interquartile range.

Analysis of patient clinical characteristics (Table 1) indicated that alogliptin had a relatively higher proportion of reports among male individuals, while the gender distribution of AEs reports for other DPP4 inhibitors was more balanced. In terms of age distribution, the median age for saxagliptin related AEs was 67 years, whereas AEs for the remaining DPP4 inhibitors primarily occurred in individuals aged 70 years and older. Geographically, the reporting sources for different DPP4 inhibitors exhibited regional patterns: reports for sitagliptin and vildagliptin were predominantly from France, those for saxagliptin and linagliptin were mainly from the United States, and reports for alogliptin were largely concentrated in Japan. Regarding clinical outcomes, all DPP4 inhibitors were associated with initial or prolonged hospitalization, and other serious/important medical event. Notably, vildagliptin showed a higher correlation with life threating outcomes (24.70%), while the proportion of mortality events observed for other DPP4 inhibitors remained at a relatively high level, ranging from 8.10% to 22.99%.

### Association signal detection at the SMQ level

**Fig 2:**
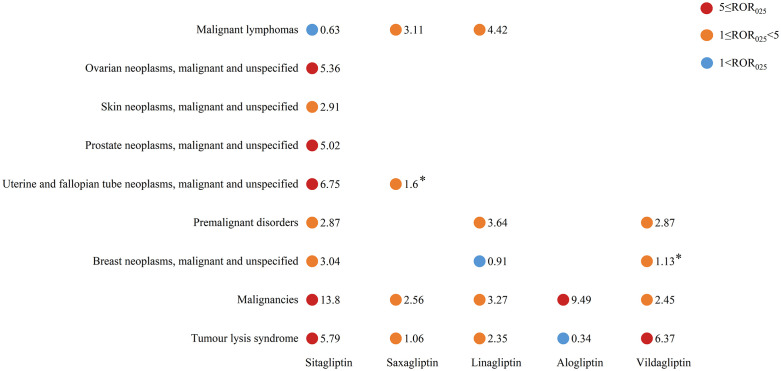
Malignancy adverse event risks of different DPP4 inhibitors at the SMQ level. Note: ROR_025_ and IC_025_ represent the lower limits of the 95% confidence intervals of ROR and IC, respectively. Signals were defined when both ROR_025_ > 1, and IC_025_ > 0. The * represents IC_025_ < 0.

Using the SMQs, we clustered AEs at the Preferred Term level into a hierarchical structure comprising nine malignancy related disease categories. As shown in Fig 2, all DPP-4 inhibitors exhibited significant malignancy risk signals, with sitagliptin demonstrating the broadest spectrum of adverse event associations. Notably, sitagliptin showed significant correlations across multiple cancer categories, particularly in tumor lysis syndrome and reproductive system malignancies. Vildagliptin was predominantly associated with tumor lysis syndrome and premalignant conditions, while linagliptin and saxagliptin shared associations with lymphoproliferative disorders. The complete statistical parameters (ROR_025_ and IC_025_ values) for each drug-class association are presented in Figs 2 and S2 Table.

### The spectrum of malignancy risks at the PT level

**Fig 3:**
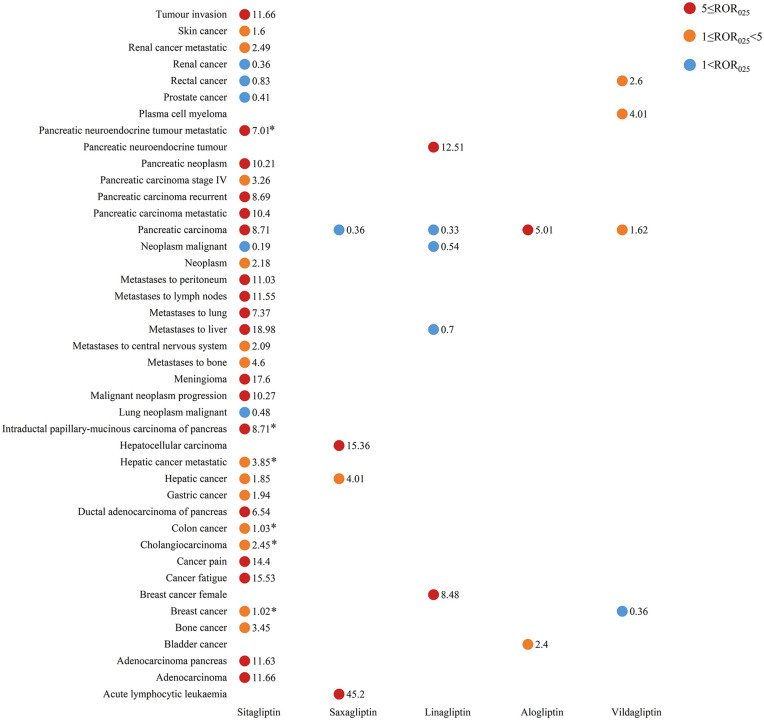
Malignancy adverse event risks of different DPP4 inhibitors at the PT level. Note: ROR_025_ and IC_025_ represent the lower limits of the 95% confidence intervals of ROR and IC, respectively. Signals were defined when both ROR_025_ > 1, and IC_025_ > 0. The * represents IC_025_ < 0.

At the PT level, we identified 35 statistically significant positive signals (ROR_025_ > 1 and IC_025_ > 0). Sitagliptin accounted for 71.43% of all signals, demonstrating the broadest spectrum of associations. These included multiple pancreatic-related events as well as metastases to liver, lymph nodes, and peritoneum. Notably, it also showed signals for meningioma and malignant neoplasm progression. Among other DPP-4 inhibitors: saxagliptin was primarily associated with acute lymphocytic leukemia and hepatocellular carcinoma, alogliptin and vildagliptin both exhibited significant signals for pancreatic carcinoma, linagliptin showed distinct associations with pancreatic neuroendocrine tumor and female breast cancer. The complete signal strength metrics (ROR_025_ and IC_025_ values) for these associations are systematically presented in Figs 3 and S3 Table, with sitagliptin showing the highest risk estimates for metastatic events among all investigated drugs.

### Sensitivity analysis

To control for the potential impact of confounding factors (such as concomitant medications), we have listed the top 10 concomitant medications in S4 Table. Based on FDA label information, we identified three commonly used co-administered drugs, including glimepiride (4.26%), sitagliptin phosphate (2.88%), and empagliflozin (2.79%), which may lead to potential cancer related AEs. Consequently, these three medications, along with age and gender, were included as confounding variables in the binary logistic regression model for analysis. Furthermore, to minimize the potential influence of different indications on the results, the analysis was limited to T2DM individuals.

As shown in Table 2, after adjusting for confounding factors, the study results indicated that sitagliptin (adjusted ROR: 4.248, 95%CI: 4.146–4.352), linagliptin (adjusted ROR: 1.391, 95%CI: 1.324–1.461), and vildagliptin (adjusted ROR: 3.204, 95%CI: 3.080–3.334) were significantly associated with malignancy AEs. By contrast, alogliptin (adjusted ROR: 0.177, 95%CI: 0.145–0.216) showed a significant negative correlation with malignancy AEs. Additionally, although the adjusted ROR for saxagliptin was 1.032, indicating a slight increase in the risk of malignancy AEs, this association did not reach statistical significance (P > 0.05), thus precluding a definitive conclusion.

**Table 2:** Sensitivity analysis of malignancy adverse events associated with DPP4 inhibitors.

Drugs	Drug-event Pairs (Exposed Cases)	Drug-event Pairs (Unexposed)	ROR (95% CI)	Adjust ROR (95%CI)
**Sitagliptin**	30,794	944,037	4.361(4.273, 4.450)	4.248(4.146, 4.352)
**Saxagliptin**	1,499	973,332	1.283(1.201, 1.370)	1.032(0.961, 1.108)*
**Linagliptin**	3,736	971,095	2.002(1.911, 2.096)	1.391(1.324, 1.461)
**Alogliptin**	111	974,720	0.249(0.204, 0.303)	0.177(0.145, 0.216)
**Vildagliptin**	9,329	965,502	3.600(3.477, 3.727)	3.204(3.080, 3.334)

Note: Adjust ROR, adjusted for age, gender and concomitant drugs (glimepiride, sitagliptin phosphate, empagliflozin) via a binary logistic regression. *p > 0.05 was not considered statistically significant.

### Time to onset of malignancy adverse event

**Fig 4:**
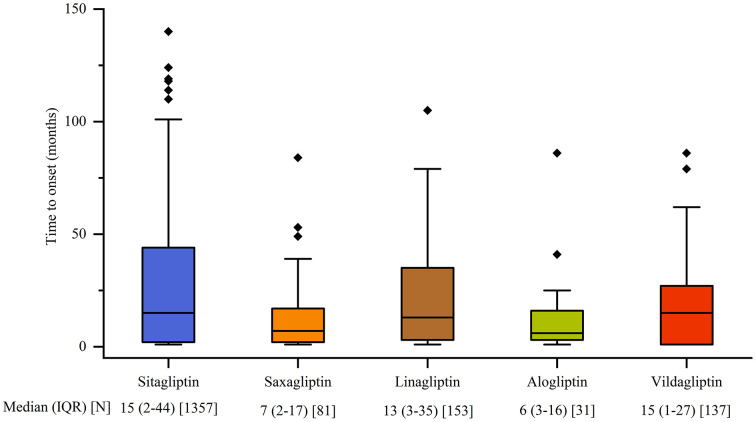
Time to onset of malignancy adverse events. Abbreviations: IQR, interquartile range; N, Number of reported cases.

Fig 4 depicts the onset time of malignancy AEs for various DPP4 inhibitors. Alogliptin demonstrated an earlier median onset time, at 6 months (IQR: 3–16 months), closely followed by saxagliptin at 7 months (IQR: 2–17 months). Conversely, the longest median onset time was observed for vildagliptin at 15 months (IQR: 1–27 months) and sitagliptin at 15 months (IQR: 2–44 months). The median times for linagliptin was 13 months (IQR: 3–35 months).

## Discussion

Diabetes and cancer, as prevalent diseases worldwide, have received increasing attention due to their interrelationship and impact on public health. Epidemiological studies have confirmed that individuals with diabetes exhibit a significantly elevated risk of developing certain types of cancer. This phenomenon may be attributed to shared pathophysiological mechanisms between the two diseases, such as chronic hyperglycemia and its associated metabolic disturbances (e.g., activation of the polyol pathway and advanced glycation end-product formation) as well as systemic inflammation and dysregulated angiogenesis mediated by factors like vascular endothelial growth factor and angiopoietin-2 [15]. A joint statement by the American Diabetes Association (ADA) and the American Cancer Society (ACS) highlights that individuals with T2DM not only have a higher incidence of cancer but also experience poorer prognoses compared to individuals without diabetes [16].

With the widespread use of DPP4 inhibitors as a novel diabetes therapeutics, their potential cancer risk has become a critical focus in clinical research. Although observational and retrospective studies have explored the association between DPP4 inhibitors and cancer incidence, the limitations in study design and data sources (e.g., short follow-up, confounding factors) have resulted in a lack of systematic evidence [17]. To identify potential pharmacovigilance signals, our study analyzed the FAERS database using quantitative disproportionality methods to evaluate the association between DPP4 inhibitors and cancer occurrence in T2DM. These findings, while hypothesis-generating, may provide a foundation for subsequent prospective research.

### Evidence from retrospective data analysis

We employed a retrospective pharmacovigilance approach to analyze six years of FAERS database records. After rigorous data cleaning and deduplication, we identified 3,764 reports of potential malignancy risks associated with DPP4 inhibitors, systematically assessing their association with malignancies.

Descriptive analysis results indicated that the reporting rates of malignancy related AEs were similar between male and female individuals. The analysis also found that middle-aged and elderly individuals showed higher susceptibility to such AEs. This observation aligns with established evidence that advanced age, along with prolonged diabetes duration, and insulin therapy, constitutes recognized risk factors for malignancy development in T2DM populations [18,19]. The potential mechanisms may involve age-related immunosenescence, accumulated metabolic dysfunction, and higher comorbidity burdens in these patient subgroups. Among different DPP4 inhibitors, alogliptin and saxagliptin showed relatively earlier onset times of malignancy. This phenomenon may stem from the small sample size and insufficient data integrity, leading to bias in median time calculations. By contrast, for other drugs with larger sample sizes, the median reporting time for AEs was approximately 14 months, a result that may be more representative. The findings of this study suggest that the long-term medication safety of middle-aged and elderly individuals should be a primary focus when using DPP4 inhibitors clinically, and larger-scale prospective studies are recommended to further validate the adverse reaction time characteristics of different DPP4 inhibitors.

Furthermore, we found that current drug labels do not explicitly indicate that DPP4 inhibitors have cancer related AEs. Although toxicological studies have shown that mouse experimental data suggest some DPP4 inhibitors have potential carcinogenicity, their clinical significance remains unclear. However, our study identified statistically significant associations between the five DPP4 inhibitors investigated and malignancies. Given the statistical significance of these pharmacovigilance signals, we evaluated whether existing experimental data could provide biological plausibility.

### Potential mechanisms and considerations for DPP4 inhibitor-associated cancer risk

While our study did not directly investigate the molecular mechanisms linking DPP-4 inhibitors to cancer risk, existing literature suggests several potential pathways that warrant consideration in interpreting our findings. The impact of DPP4 inhibitors on malignancy biology is multifaceted, involving multiple signaling pathways and the regulation of the tumor microenvironment. Firstly, DPP4 inhibitors reduce the degradation of various bioactive molecules (such as growth factors, cytokines, chemokines, and neuropeptides) by inhibiting the enzymatic activity of DPP4, leading to a significant increase in the levels of these substrates in the tumor microenvironment [20,21]. The accumulation of these substrates can promote tumor progression through various mechanisms, including enhancing the metastatic potential of tumor cells, inducing chemotherapy resistance, and promoting tumor growth [17].

Specifically, DPP4 inhibitors significantly increase the bioavailability of C-X-C motif chemokine 12 (CXCL12) by inhibiting its degradation. Elevated CXCL12 binds to its receptor C-X-C receptor 4 (CXCR4), activating the CXCL12/CXCR4/mTOR (mammalian target of rapamycin) signaling pathway, which in turn promotes tumor cell proliferation, migration, and invasion [22,23]. Additionally, the activation of this signaling pathway induces epithelial-mesenchymal transition (EMT), a process that not only enhances the metastatic ability of tumor cells but also increases chemotherapy resistance by upregulating the expression of ATP-binding cassette (ABC) transporters [24]. Meanwhile, the activation of the mTOR signaling pathway further reinforces the EMT process, creating a positive feedback loop that exacerbates tumor invasiveness and metastatic potential [25].

Beyond its effects on the CXCL12/CXCR4/mTOR signaling pathway, DPP4 inhibitors may also regulate tumor progression by altering the tumor microenvironment and immune system function. For example, DPP4 inhibitors may affect the infiltration and function of immune cells by increasing the levels of certain chemokines, thereby altering the immunosuppressive state of the tumor microenvironment. Such changes in the immune microenvironment may further influence tumor growth and metastasis [26,27]. Studies have demonstrated that incretin-based therapies can induce pancreatic tissue expansion. In exocrine tissues, this manifests as increased proliferation and dysplasia. In endocrine tissues, α-cell hyperplasia may progress to neuroendocrine tumors. This suggests that DPP4 inhibitors may have potential tumor-promoting effects in certain contexts [28].

In summary, the impact of DPP-4 inhibitors on tumor biology appears context-dependent, with preclinical evidence supporting both pro- and anti-tumor effects and further suggesting that these effects are more consistently related to tumor progression than to its initiation [29,30]. While our analysis identified elevated signals for metastatic cancers (e.g., liver metastases [ROR_025_:18.98, IC_025_:3.33], pancreatic adenocarcinoma [ROR_025_:10.40, IC_025_:2.82]), other studies demonstrate sitagliptin may suppress colorectal cancer metastasis in vitro [31]. These discrepancies may reflect tissue-specific mechanisms or model system differences. In light of the elevated baseline cancer risk in the T2DM population, the disproportionality signals observed in our FAERS analysis could reflect a potential exacerbation of cancer risk or progression, rather than direct carcinogenesis. As a pharmacovigilance study, our analysis cannot distinguish between these possibilities; however, the observed signals emphasize the need for integrated clinical and mechanistic investigations to determine whether DPP4 inhibitors influence cancer incidence or outcomes in patients with T2DM.

### Limitation

The relationship between DPP4 inhibitors and tumor incidence is influenced by multiple complex factors. Firstly, the retrospective study design lacks a control group, which inherently limits the ability to accurately assess the impact of antidiabetic drugs on cancer risk. Moreover, although we adjusted for potential confounders—such as indication restrictions, age, sex, and concomitant medications associated with tumor risk through sensitivity analyses, residual biases (e.g., genetic predisposition, lifestyle differences, pre-existing malignancies) may remain uncontrolled. Therefore, prospective studies or randomized controlled trials are needed to further validate any causal association [32]. Secondly, the sample size of individuals using DPP4 inhibitors alone is relatively limited, while individuals receiving combination therapy with DPP4 inhibitors often exhibit poor glycemic control. This metabolic disorder may potentially increase the risk of cancer development [33]. Notwithstanding these constraints, this pharmacovigilance analysis, drawing on an initial dataset of over 10 million real‑world reports and a final analytical cohort of 673,768 T2DM patients, provides valuable hypothesis‑generating evidence. To establish a definitive safety profile regarding cancer risk, the findings warrant validation through prospective studies or randomized controlled trials with longer follow‑up.

## Conclusion

Our retrospective analysis found that while existing drug labels do not explicitly indicate malignancy related AEs for DPP4 inhibitors, and the clinical significance of potential carcinogenicity in mouse studies remains unclear, all five DPP4 inhibitors discussed showed statistically significant signals associated with malignancies. This finding provides important evidence for clinical drug safety, suggesting that monitoring of malignancy related AEs should be strengthened during clinical use, particularly for middle-aged and elderly individuals as well as high-risk populations. The research outcomes may contribute to the development of individualized treatment strategies, provide insights for optimizing drug vigilance systems, and guide further mechanistic studies.

## Supporting information

S1 Table Major algorithms used for pharmacovigilance analysis. (DOCX)

S2 Table Signal strength for SMQ. (DOCX)

S3 Table Signal strength for Preferred Term (PT). (DOCX)

S4 Table Top 10 concomitant drugs with DPP4 inhibitors. (DOCX)

## References

[1] Sohn M, Nam S, Nauck MA, Lim S. Long-term comparison of renal and metabolic outcomes after sodium-glucose co-transporter 2 inhibitor or glucagon-like peptide-1 receptor agonist therapy in type 2 diabetes. BMC Med. 2024;22(1):273. doi: 10.1186/s12916-024-03483-z 38956548 PMC11218058

[2] Deacon CF. Dipeptidyl peptidase 4 inhibitors in the treatment of type 2 diabetes mellitus. Nat Rev Endocrinol. 2020;16(11):642–53. doi: 10.1038/s41574-020-0399-8 32929230

[3] Butler PC, Elashoff M, Elashoff R, Gale EAM. A critical analysis of the clinical use of incretin-based therapies: are the GLP-1 therapies safe? Diabetes Care. 2013;36(7):2118–25. doi: 10.2337/dc12-2713 23645885 PMC3687282

[4] Stoian AP, Sachinidis A, Stoica RA, Nikolic D, Patti AM, Rizvi AA. The efficacy and safety of dipeptidyl peptidase-4 inhibitors compared to other oral glucose-lowering medications in the treatment of type 2 diabetes. Metabolism. 2020;109:154295. doi: 10.1016/j.metabol.2020.154295 32553739

[5] Nagel AK, Ahmed-Sarwar N, Werner PM, Cipriano GC, Van Manen RP, Brown JE. Dipeptidyl peptidase-4 inhibitor-associated pancreatic carcinoma: a review of the FAERS database. Ann Pharmacother. 2016;50(1):27–31. doi: 10.1177/1060028015610123 26497885

[6] Butler AE, Campbell-Thompson M, Gurlo T, Dawson DW, Atkinson M, Butler PC. Marked expansion of exocrine and endocrine pancreas with incretin therapy in humans with increased exocrine pancreas dysplasia and the potential for glucagon-producing neuroendocrine tumors. Diabetes. 2013;62(7):2595–604. doi: 10.2337/db12-1686 23524641 PMC3712065

[7] Abrahami D, Douros A, Yin H, Yu OH, Faillie J-L, Montastruc F, et al. Incretin based drugs and risk of cholangiocarcinoma among patients with type 2 diabetes: population based cohort study. BMJ. 2018;363:k4880. doi: 10.1136/bmj.k4880 30518618 PMC6278586

[8] Niazmand A, Nedaeinia R, Vatandoost N, Jafarpour S, Safabakhsh S, Kolahdouz M, et al. The impacts of dipeptidyl- peptidase 4 (DPP-4) inhibitors on common female malignancies: a systematic review. Gene. 2024;927:148659. doi: 10.1016/j.gene.2024.148659 38866262

[9] Pinto LC, Rados DV, Barkan SS, Leitão CB, Gross JL. Dipeptidyl peptidase-4 inhibitors, pancreatic cancer and acute pancreatitis: a meta-analysis with trial sequential analysis. Sci Rep. 2018;8(1):782. doi: 10.1038/s41598-017-19055-6 29335646 PMC5768864

[10] Dicembrini I, Nreu B, Montereggi C, Mannucci E, Monami M. Risk of cancer in patients treated with dipeptidyl peptidase-4 inhibitors: an extensive meta-analysis of randomized controlled trials. Acta Diabetol. 2020;57(6):689–96. doi: 10.1007/s00592-020-01479-8 31955260

[11] Xiong W, Li Y, Hu L, He G, Huang J. Risks of malignancies related to disease-modifying antirheumatic drugs in rheumatoid arthritis: a pharmacovigilance analysis using the FAERS database. Front Pharmacol. 2024;15:1458500. doi: 10.3389/fphar.2024.1458500 39605908 PMC11598350

[12] Salah S, Kerob D, Pages Laurent C, Lacouture M, Sibaud V. Evaluation of anticancer therapy-related dermatologic adverse events: insights from Food and Drug Administration’s Adverse Event Reporting System dataset. J Am Acad Dermatol. 2024;91(5):863–71. doi: 10.1016/j.jaad.2024.07.1456 39038557

[13] Available from: https://www.fda.gov/drugs/fdas-adverse-event-reporting-system-faers/fda-adverse-event-reporting-system-faers-latest-quarterly-data-files

[14] Tyagi S, Kumar A. Safety of immune checkpoint inhibitors: an updated comprehensive disproportionality analysis and meta-analysis. Crit Rev Oncol Hematol. 2024;200:104398. doi: 10.1016/j.critrevonc.2024.104398 38810844

[15] Chang W-C, Hsieh T-C, Hsu W-L, Chang F-L, Tsai H-R, He M-S. Diabetes and further risk of cancer: a nationwide population-based study. BMC Med. 2024;22(1):214. doi: 10.1186/s12916-024-03430-y 38807177 PMC11134680

[16] Williams R, Karuranga S, Malanda B, Saeedi P, Basit A, Besançon S, et al. Global and regional estimates and projections of diabetes-related health expenditure: results from the International Diabetes Federation Diabetes Atlas, 9th edition. Diabetes Res Clin Pract. 2020;162:108072. doi: 10.1016/j.diabres.2020.108072 32061820

[17] Kawakita E, Koya D, Kanasaki K. CD26/DPP-4: type 2 diabetes drug target with potential influence on cancer biology. Cancers (Basel). 2021;13(9):2191. doi: 10.3390/cancers13092191 34063285 PMC8124456

[18] Chou C-L, Juan S-H, Li C-H, Chen H-H, Kao C-C, Chen L-Y, et al. Association between DPP-4 inhibitors and events of colorectal and liver cancers in patients with diabetes receiving second-line agents: a nested case-control study. Front Oncol. 2022;12:840142. doi: 10.3389/fonc.2022.840142 35600378 PMC9120816

[19] Scarton L, Jo A, Xie Z, O’Neal LJ, Munoz Pena JM, George TJ, et al. Examining the relationship between metformin dose and cancer survival: a SEER-Medicare analysis. PLoS One. 2022;17(10):e0275681. doi: 10.1371/journal.pone.0275681 36260549 PMC9581409

[20] Yu DMT, Yao T-W, Chowdhury S, Nadvi NA, Osborne B, Church WB, et al. The dipeptidyl peptidase IV family in cancer and cell biology. FEBS J. 2010;277(5):1126–44. doi: 10.1111/j.1742-4658.2009.07526.x 20074209

[21] Moffitt LR, Bilandzic M, Wilson AL, Chen Y, Gorrell MD, Oehler MK, et al. Hypoxia regulates DPP4 expression, proteolytic inactivation, and shedding from ovarian cancer cells. Int J Mol Sci. 2020;21(21):8110. doi: 10.3390/ijms21218110 33143089 PMC7672561

[22] Yang F, Takagaki Y, Yoshitomi Y, Ikeda T, Li J, Kitada M, et al. Inhibition of dipeptidyl peptidase-4 accelerates epithelial-mesenchymal transition and breast cancer metastasis via the CXCL12/CXCR4/mTOR axis. Cancer Res. 2019;79(4):735–46. doi: 10.1158/0008-5472.CAN-18-0620 30584072

[23] He L, Zhang T, Sun W, Qin Y, Wang Z, Dong W, et al. The DPP-IV inhibitor saxagliptin promotes the migration and invasion of papillary thyroid carcinoma cells via the NRF2/HO1 pathway. Med Oncol. 2020;37(11):97. doi: 10.1007/s12032-020-01419-0 33001278

[24] Begicevic R-R, Falasca M. ABC Transporters in cancer stem cells: beyond chemoresistance. Int J Mol Sci. 2017;18(11):2362. doi: 10.3390/ijms18112362 29117122 PMC5713331

[25] Katsuno Y, Meyer DS, Zhang Z, Shokat KM, Akhurst RJ, Miyazono K, et al. Chronic TGF-β exposure drives stabilized EMT, tumor stemness, and cancer drug resistance with vulnerability to bitopic mTOR inhibition. Sci Signal. 2019;12(570):eaau8544. doi: 10.1126/scisignal.aau8544 30808819 PMC6746178

[26] Shin SY, Nam J-S, Lim Y, Lee YH. TNFα-exposed bone marrow-derived mesenchymal stem cells promote locomotion of MDA-MB-231 breast cancer cells through transcriptional activation of CXCR3 ligand chemokines. J Biol Chem. 2010;285(40):30731–40. doi: 10.1074/jbc.M110.128124 20650898 PMC2945567

[27] Johnson DC, Taabazuing CY, Okondo MC, Chui AJ, Rao SD, Brown FC, et al. DPP8/DPP9 inhibitor-induced pyroptosis for treatment of acute myeloid leukemia. Nat Med. 2018;24(8):1151–6. doi: 10.1038/s41591-018-0082-y 29967349 PMC6082709

[28] Gelling RW, Du XQ, Dichmann DS, Romer J, Huang H, Cui L, et al. Lower blood glucose, hyperglucagonemia, and pancreatic alpha cell hyperplasia in glucagon receptor knockout mice. Proc Natl Acad Sci U S A. 2003;100(3):1438–43. doi: 10.1073/pnas.0237106100 12552113 PMC298791

[29] Tschöp MH, Stumvoll M, Ristow M. Opposing effects of antidiabetic interventions on malignant growth and metastasis. Cell Metab. 2016;23(6):959–60. doi: 10.1016/j.cmet.2016.05.017 27304493

[30] Nishina S, Yamauchi A, Kawaguchi T, Kaku K, Goto M, Sasaki K, et al. Dipeptidyl peptidase 4 inhibitors reduce hepatocellular carcinoma by activating lymphocyte chemotaxis in mice. Cell Mol Gastroenterol Hepatol. 2018;7(1):115–34. doi: 10.1016/j.jcmgh.2018.08.008 30510994 PMC6260362

[31] Varela-Calviño R, Rodríguez-Quiroga M, Dias Carvalho P, Martins F, Serra-Roma A, Vázquez-Iglesias L, et al. The mechanism of sitagliptin inhibition of colorectal cancer cell lines’ metastatic functionalities. IUBMB Life. 2021;73(5):761–73. doi: 10.1002/iub.2454 33615655

[32] Liu W-H, Hu H-M, Li C, Shi Q, Liu C-H, Liu A-X, et al. Real-world study of adverse events associated with triptan use in migraine treatment based on the U.S. Food and Drug Administration (FDA) adverse event reporting system (FAERS) database. J Headache Pain. 2024;25(1):206. doi: 10.1186/s10194-024-01913-0 39587512 PMC11587596

[33] Giri B, Dey S, Das T, Sarkar M, Banerjee J, Dash SK. Chronic hyperglycemia mediated physiological alteration and metabolic distortion leads to organ dysfunction, infection, cancer progression and other pathophysiological consequences: an update on glucose toxicity. Biomed Pharmacother. 2018;107:306–28. doi: 10.1016/j.biopha.2018.07.157 30098549

