# Supplementary material for: Cancer risk associated with DPP4 inhibitors in type 2 diabetes: A pharmacovigilance analysis of the FDA Adverse Event Reporting System (FAERS)
Source: PLoS One. 2026 Mar 20;21(3):e0345281. doi: 10.1371/journal.pone.0345281 (PMC13004328; doi:10.1371/journal.pone.0345281)
Supplement: S1 Table — (DOCX) [file pone.0345281.s001.docx]

# S1 Table. Major algorithms used for pharmacovigilance analysis

| **Algorithms** | **Equation** | **Criteria** |
| --- | --- | --- |
| ROR | ROR=ad/b/c  95%CI=eln(ROR)±1.96(1/a+1/b+1/c+1/d)^0.5 | ROR_025_ > 1 |
| IC | IC=log_2_a(a+b+c+d)(a+c)(a+b)  95%CI= E(IC) ± 2V(IC)^0.5 | IC_025_ > 0 |

Abbreviations: a, number of reports containing both the target drug and target adverse event; b, number of reports containing other adverse event of the target drug; c, number of reports containing the target adverse event of other drugs; d, number of reports containing other drugs and other adverse event. 95%CI, 95% confidence interval; N, the number of reports; E(IC), the IC expectations; V(IC), the variance of IC.
