# Supplementary material for: Cancer risk associated with DPP4 inhibitors in type 2 diabetes: A pharmacovigilance analysis of the FDA Adverse Event Reporting System (FAERS)
Source: PLoS One. 2026 Mar 20;21(3):e0345281. doi: 10.1371/journal.pone.0345281 (PMC13004328; doi:10.1371/journal.pone.0345281)
Supplement: S2 Table — (DOCX) [file pone.0345281.s002.docx]

# S2 Table. Signal strength for SMQ.

| Drugs | SMQ | Case(n) | ROR(95%CI) | IC(95%CI) |
| --- | --- | --- | --- | --- |
| Sitagliptin | Tumour lysis syndrome | 1480 | 6.12(5.79-6.47) | 2.41(2.32-2.48) |
| Sitagliptin | Malignancies | 1046 | 14.76(13.8-15.79) | 3.59(3.48-3.67) |
| Sitagliptin | Breast neoplasms, malignant and unspecified | 31 | 4.36(3.04-6.26) | 2.07(1.41-2.45) |
| Sitagliptin | Premalignant disorders | 27 | 4.22(2.87-6.2) | 2.02(1.31-2.43) |
| Sitagliptin | Uterine and fallopian tube neoplasms, malignant and unspecified | 27 | 10.04(6.75-14.95) | 3.19(2.25-3.39) |
| Sitagliptin | Prostate neoplasms, malignant and unspecified | 16 | 8.39(5.02-14) | 2.95(1.74-3.2) |
| Sitagliptin | Skin neoplasms, malignant and unspecified | 13 | 5.08(2.91-8.88) | 2.28(1.13-2.72) |
| Sitagliptin | Ovarian neoplasms, malignant and unspecified | 5 | 13.73(5.36-35.18) | 3.59(0.81-3.34) |
| Saxagliptin | Tumour lysis syndrome | 76 | 1.34(1.06-1.68) | 0.41(0.07-0.74) |
| Saxagliptin | Malignancies | 58 | 3.32(2.56-4.31) | 1.7(1.27-2.03) |
| Saxagliptin | Malignant lymphomas | 3 | 9.78(3.11-30.71) | 3.26(0.14-3.07) |
| Linagliptin | Tumour lysis syndrome | 166 | 2.75(2.35-3.21) | 1.4(1.16-1.62) |
| Linagliptin | Malignancies | 78 | 4.1(3.27-5.14) | 2(1.61-2.27) |
| Linagliptin | Premalignant disorders | 10 | 6.81(3.64-12.74) | 2.74(1.26-3.02) |
| Linagliptin | Malignant lymphomas | 4 | 11.97(4.42-32.36) | 3.54(0.58-3.21) |
| Alogliptin | Malignancies | 43 | 12.96(9.49-17.7) | 3.58(2.81-3.71) |
| Vildagliptin | Tumour lysis syndrome | 552 | 6.97(6.37-7.63) | 2.61(2.47-2.73) |
| Vildagliptin | Malignancies | 84 | 3.05(2.45-3.79) | 1.58(1.23-1.86) |
| Vildagliptin | Premalignant disorders | 11 | 5.21(2.87-9.47) | 2.36(1.09-2.77) |

Note: Only significant signals are recorded.
