# Supplementary material for: Cancer risk associated with DPP4 inhibitors in type 2 diabetes: A pharmacovigilance analysis of the FDA Adverse Event Reporting System (FAERS)
Source: PLoS One. 2026 Mar 20;21(3):e0345281. doi: 10.1371/journal.pone.0345281 (PMC13004328; doi:10.1371/journal.pone.0345281)
Supplement: S3 Table — (DOCX) [file pone.0345281.s003.docx]

# S3 Table. Signal strength for Preferred Term (PT).

| Drugs | PT | Case(n) | ROR(95%CI) | IC(95%CI) |
| --- | --- | --- | --- | --- |
| Sitagliptin | Pancreatic carcinoma | 111 | 10.79(8.71-13.36) | 3.07(2.67-3.28) |
| Sitagliptin | Metastases to liver | 91 | 24.85(18.98-32.53) | 3.89(3.33-4.04) |
| Sitagliptin | Pancreatic carcinoma metastatic | 90 | 13.27(10.4-16.93) | 3.3(2.82-3.5) |
| Sitagliptin | Adenocarcinoma pancreas | 42 | 16.82(11.63-24.31) | 3.54(2.7-3.72) |
| Sitagliptin | Pancreatic neoplasm | 28 | 15.97(10.21-24.99) | 3.49(2.43-3.66) |
| Sitagliptin | Malignant neoplasm progression | 19 | 17.86(10.27-31.06) | 3.6(2.2-3.7) |
| Sitagliptin | Metastases to lymph nodes | 17 | 21.11(11.55-38.58) | 3.75(2.17-3.78) |
| Sitagliptin | Metastases to lung | 15 | 13.37(7.37-24.26) | 3.31(1.84-3.48) |
| Sitagliptin | Meningioma | 11 | 42.48(17.6-102.54) | 4.3(1.87-3.96) |
| Sitagliptin | Metastases to peritoneum | 10 | 24.83(11.03-55.9) | 3.9(1.65-3.74) |
| Sitagliptin | Cancer pain | 8 | 39.72(14.4-109.54) | 4.25(1.43-3.84) |
| Sitagliptin | Neoplasm | 8 | 4.56(2.18-9.52) | 2.05(0.58-2.63) |
| Sitagliptin | Bone cancer | 7 | 7.85(3.45-17.82) | 2.72(0.82-3.06) |
| Sitagliptin | Hepatic cancer | 7 | 4.05(1.85-8.87) | 1.9(0.38-2.54) |
| Sitagliptin | Gastric cancer | 6 | 4.53(1.94-10.61) | 2.04(0.33-2.66) |
| Sitagliptin | Metastases to bone | 6 | 11.58(4.6-29.18) | 3.16(0.81-3.28) |
| Sitagliptin | Adenocarcinoma | 5 | 43.43(11.66-161.76) | 4.31(0.74-3.72) |
| Sitagliptin | Pancreatic carcinoma stage IV | 5 | 8.69(3.26-23.15) | 2.84(0.49-3.1) |
| Sitagliptin | Skin cancer | 5 | 4.04(1.6-10.2) | 1.9(0.09-2.59) |
| Sitagliptin | Tumour invasion | 5 | 43.43(11.66-161.76) | 4.31(0.74-3.72) |
| Sitagliptin | Cancer fatigue | 4 | 138.97(15.53-1243.48) | 4.84(0.35-3.85) |
| Sitagliptin | Ductal adenocarcinoma of pancreas | 4 | 23.16(6.54-82.09) | 3.84(0.38-3.49) |
| Sitagliptin | Metastases to central nervous system | 4 | 6.04(2.09-17.47) | 2.4(0.09-2.89) |
| Sitagliptin | Pancreatic carcinoma recurrent | 4 | 34.74(8.69-138.93) | 4.16(0.39-3.61) |
| Sitagliptin | Renal cancer metastatic | 4 | 7.31(2.49-21.5) | 2.64(0.16-3) |
| Saxagliptin | Acute lymphocytic leukaemia | 6 | 130.34(45.2-375.91) | 6.22(1.37-4.01) |
| Saxagliptin | Hepatic cancer | 4 | 11.03(4.01-30.31) | 3.38(0.51-3.18) |
| Saxagliptin | Hepatocellular carcinoma | 4 | 46.31(15.36-139.62) | 5.2(0.72-3.61) |
| Linagliptin | Breast cancer female | 4 | 24.33(8.48-69.74) | 4.41(0.67-3.46) |
| Linagliptin | Pancreatic neuroendocrine tumour | 4 | 37.21(12.51-110.64) | 4.92(0.7-3.57) |
| Alogliptin | Pancreatic carcinoma | 6 | 11.27(5.01-25.31) | 3.46(1.07-3.28) |
| Alogliptin | Bladder cancer | 3 | 7.49(2.4-23.4) | 2.89(0.05-2.96) |
| Vildagliptin | Pancreatic carcinoma | 12 | 2.87(1.62-5.09) | 1.49(0.49-2.11) |
| Vildagliptin | Plasma cell myeloma | 4 | 11.24(4.01-31.46) | 3.36(0.48-3.2) |
| Vildagliptin | Rectal cancer | 3 | 8.43(2.6-27.28) | 2.98(0.02-3.03) |

Note: Only significant signals are recorded.
