# Supplementary material for: Cancer risk associated with DPP4 inhibitors in type 2 diabetes: A pharmacovigilance analysis of the FDA Adverse Event Reporting System (FAERS)
Source: PLoS One. 2026 Mar 20;21(3):e0345281. doi: 10.1371/journal.pone.0345281 (PMC13004328; doi:10.1371/journal.pone.0345281)
Supplement: S4 Table — (DOCX) [file pone.0345281.s004.docx]

# S4 Table. Top 10 concomitant drugs with DPP4 inhibitors

| **Concomitant drugs** | **N (%)** | **AEs (FDA label information)** |
| --- | --- | --- |
| Metformin Hydrochloride | 4038（28.7%） | Not common |
| Insulin Glargine | 1137（8.08%） | Not common |
| Insulin Nos | 755（5.37%） | Not common |
| Glimepiride | 599（4.26%） | In mice, administration of glimepiride for 24 months resulted in an increase in benign pancreatic adenoma formation |
| Gliclazide | 586（4.16%） | Carcinogenicity studies have not yet been conducted. |
| Insulin Aspart | 475（3.38%） | Not common |
| Glipizide | 419（2.98%） | Not common |
| Sitagliptin Phosphate | 405（2.88%） | A two-year carcinogenicity study was conducted in male and female rats given oral doses of sitagliptin of 50, 150, and 500 mg/kg/day. There was an increased incidence of combined liver adenoma/carcinoma in males and females and of liver carcinoma in females at 500 mg/kg. |
| Empagliflozin | 393（2.79%） | In CD-1 mice and Wistar rats. In male rats, hemangiomas of the mesenteric lymph node were increased significantly at 700 mg/kg/day or approximately 42 times the exposure from a 25 mg clinical dose. Renal tubule adenomas and carcinomas were observed in male mice at 1,000 mg/kg/day, which is approximately 45 times the exposure of the maximum clinical dose of 25 mg. |
